# Dr. John W. Bickford

**Published:** 1911-03

**Authors:** 


Dr. John W. Bickford, a graduate of the University of Buffalo (1881), died at his home in Lockport, N. Y., January 31, 1911, from cerebral hemorrhage, aged 62 years. He was at one time a coroner of Niagara County, and health officer of Lockport. He had been physician to the county jail since 1908.

